# Severe Orthopnoea With Positional Paradoxical Breathing: A Response to Continuous Positive Airway Pressure

**DOI:** 10.7759/cureus.106849

**Published:** 2026-04-11

**Authors:** Julian V Jakobi, Maciej Paciorkowski, Kevin Graf, Martin H Brutsche

**Affiliations:** 1 Department of Internal Medicine, Kantonsspital St. Gallen, St. Gallen, CHE; 2 Department of Pulmonology, Kantonsspital St. Gallen, St. Gallen, CHE; 3 Department of Neurology, Kantonsspital St. Gallen, St. Gallen, CHE

**Keywords:** continuous positive airway pressure, diaphragmatic dysfunction, hypomagnesaemia, hypoventilation, nocturnal hypoxaemia, orthopnoea, paradoxical breathing, phrenic neuropathy, respiratory muscle weakness, ventilatory pump failure

## Abstract

A 54-year-old man presented with an abrupt onset disabling orthopnoea preventing supine sleep for several months. Cardiopulmonary imaging excluded heart failure, pulmonary embolism, interstitial lung disease, and upper airway obstruction. Pulmonary function testing demonstrated restrictive ventilatory impairment, and arterial blood gas revealed hypercapnia suggestive of hypoventilation. Neurological examination in the supine position showed paradoxical breathing with inspiratory thoracic expansion and inward abdominal movement, raising suspicion of diaphragmatic dysfunction. Sleep study demonstrated moderate obstructive sleep apnoea with an apnoea-hypopnoea index (AHI) of 25 events per hour and disproportionate nocturnal hypoxaemia (mean oxygen saturation 84%, nadir 65%). Profound hypomagnesaemia with associated hypokalaemia was identified and corrected. Computed tomography (CT) imaging showed a subtle elevation of the left hemidiaphragm and documented marked dyspnoea during supine acquisition. Respiratory muscle testing demonstrated severe inspiratory muscle weakness consistent with ventilatory pump limitation. Nocturnal continuous positive airway pressure (CPAP) resulted in rapid symptomatic improvement, allowing recumbent sleep. Despite extensive in-hospital evaluation, the underlying cause of orthopnoea remained undetermined. Structured differential diagnosis of progressive diaphragmatic weakness included cervical myelopathy, phrenic neuropathy, neuromuscular junction disorder, and primary myopathy. Further outpatient evaluation was planned but could not be completed because of the patient’s unexpected death from acute myocardial infarction one month after discharge. This case highlights the importance of considering ventilatory pump failure when orthopnoea is severe and cardiopulmonary evaluation is unrevealing.

## Introduction

Orthopnoea is a clinically important symptom classically associated with cardiopulmonary disease, but when standard cardiopulmonary investigations remain unrevealing, alternative mechanisms should be considered, including diaphragmatic dysfunction [1], neuromuscular disease [2], obesity-related ventilatory limitation [3], and sleep-related hypoventilation [4]. Diaphragmatic weakness may cause immediate dyspnoea upon assuming the supine position and can present with paradoxical breathing [1]. Severe electrolyte disturbances, including hypomagnesaemia and hypokalaemia [1], may contribute to diaphragmatic dysfunction. Obesity-related expiratory flow limitation has also been described as a contributor to orthopnoea in selected patients [3]. In addition, endocrine causes such as primary aldosteronism may lead to profound hypokalaemia and associated muscle weakness [5,6]. Severe hypokalaemia can lead to acute neuromuscular weakness and paralysis [5], while electrolyte disturbances, including hypocalcaemia, may also produce respiratory symptoms due to laryngospasm or stridor [7]. We report a case of disabling orthopnoea with positional paradoxical breathing and profound nocturnal desaturation in which continuous positive airway pressure (CPAP) provided rapid symptomatic relief despite the absence of a definitive diagnosis.

## Case presentation

A 54-year-old man with arterial hypertension and gastro-oesophageal reflux disease, treated with a proton pump inhibitor, developed sudden-onset dyspnoea when lying flat four months prior to admission. The orthopnoea was immediate and disabling from onset, preventing supine sleep, and he slept exclusively in a seated position. He denied exertional dyspnoea, chest pain, or peripheral oedema but reported chronic productive cough. Weight had decreased voluntarily from 125 kg to 95 kg over one year. He was an active smoker (approximately 40 pack-years) and reported occasional alcohol use. Medication included perindopril, indapamide, bisoprolol, and esomeprazole.

Vital signs were stable, with a resting oxygen saturation of 95% on room air. Supine positioning provoked immediate dyspnoea. Cardiovascular examination was unremarkable, and breath sounds were diminished bibasally.

**Figure 1:**
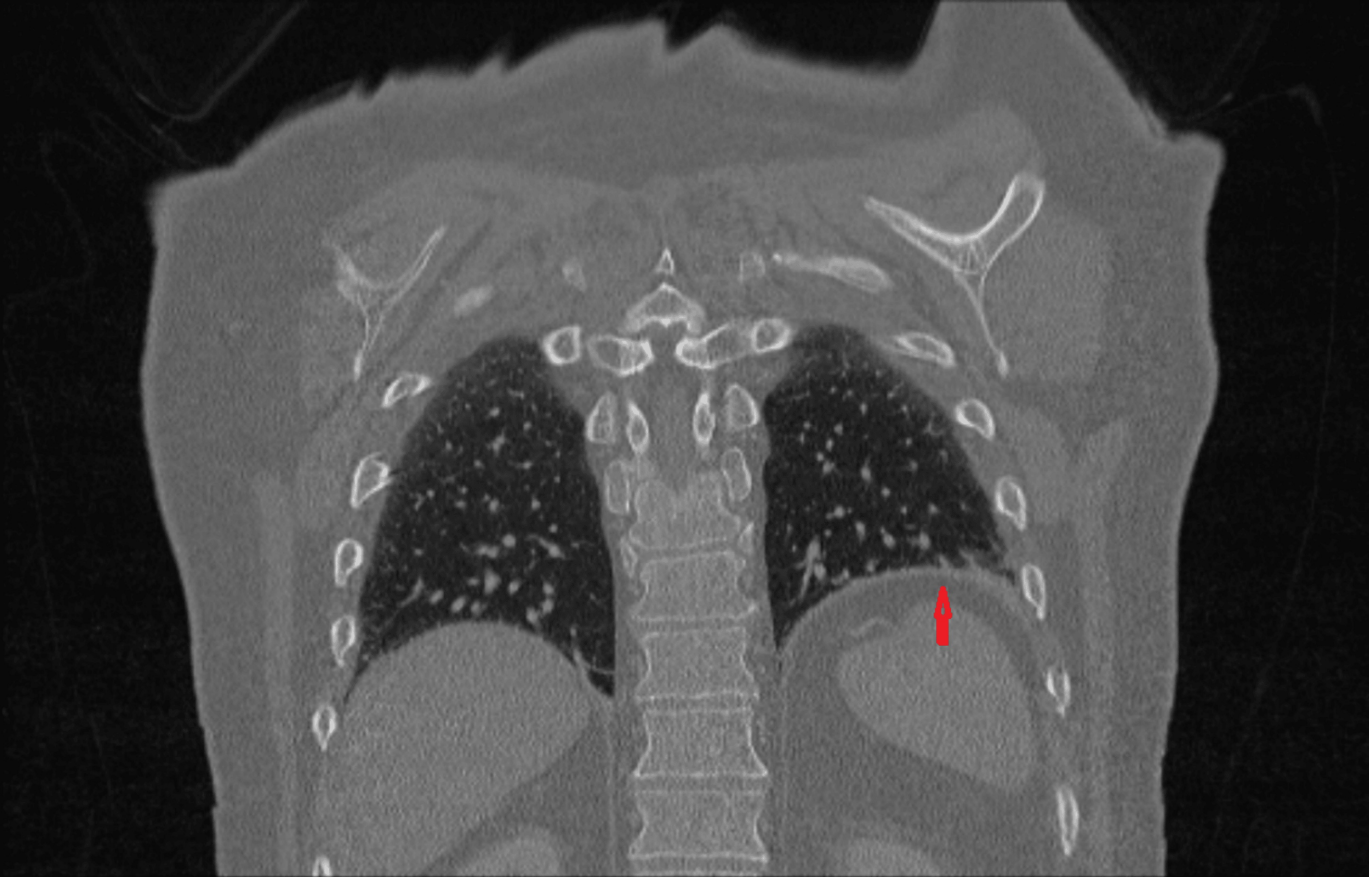
Chest CT demonstrating subtle elevation of the left hemidiaphragm without evidence of pulmonary embolism, interstitial lung disease, or airway obstruction. CT, computed tomography.

Computed tomography (CT) of the chest and abdomen showed no pulmonary embolism, interstitial lung disease, tracheal obstruction, or malignancy. Subtle elevation of the left hemidiaphragm was noted. There was pronounced dyspnoea during supine positioning for image acquisition. An incidental left adrenal lesion measuring approximately 1 cm was identified (Figure 1).

Laboratory tests revealed profound hypomagnesaemia below measurable limits <0.2 mmol/L (reference range 0.7-1.1 mmol/L) with associated moderate hypokalaemia 2.9 mmol/L (reference range 3.5-5.1 mmol/L). Electrolyte history documented recurrent hypokalaemia for several months prior to admission (laboratory results from the general practitioner). During the hospital stay, the patient developed acute diarrhoea, considered most likely due to self-limited enteritis. Multiplex polymerase chain reaction (PCR) testing for common gastrointestinal viral pathogens (including adenovirus, astrovirus, norovirus, rotavirus, and sapovirus) was negative. While this episode may have aggravated the severe hypomagnesaemia during admission, it was considered temporally distinct from the recurrent hypokalaemia already documented before hospitalisation.

Electrocardiography showed sinus rhythm with mild sinus tachycardia and no acute pathology.

**Figure 2:**
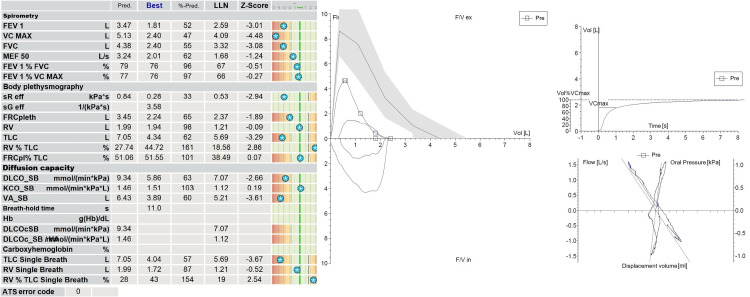
Pulmonary function testing including body plethysmography (A) Spirometry showing reduced FEV1 (52% predicted) and FVC (55% predicted) with preserved FEV1/FVC ratio (76%), consistent with a restrictive ventilatory pattern. (B) Flow-volume and volume-time curves showing technically adequate manoeuvres. Body plethysmography confirms reduced total lung capacity and alveolar volume. Abbreviations: FEV1, forced expiratory volume in 1 second; VCmax, maximal vital capacity; FVC, forced vital capacity; MEF50, maximal expiratory flow at 50% of FVC; FEV1%FVC, FEV1/FVC ratio; FEV1%VCmax, FEV1/VCmax ratio; sReff, effective specific airway resistance; sGeff, effective specific airway conductance; FRCpleth, plethysmographic functional residual capacity; RV, residual volume; TLC, total lung capacity; RV%TLC, RV/TLC ratio; FRCpl%TLC, FRCpleth/TLC ratio; DLCO_SB, single-breath diffusing capacity for carbon monoxide; KCO_SB, carbon monoxide transfer coefficient; VA_SB, single-breath alveolar volume; Hb, haemoglobin; DLCOcSB, haemoglobin-corrected DLCO_SB; DLCOc_SB/VA, haemoglobin-corrected KCO_SB; TLC Single Breath, single-breath TLC; RV Single Breath, single-breath RV; RV%TLC Single Breath, single-breath RV/TLC ratio; Pred., predicted; Best, best value; %-Pred., percent predicted; LLN, lower limit of normal; Z-Score, z score relative to the reference population; F/V ex, expiratory flow-volume curve; F/V in, inspiratory flow-volume curve; Vol, volume; Pre, pre-bronchodilator; Vol%VCmax, volume as percent of VCmax. Units: L, litres; s, seconds; L/s, litres/second; kPa·s, kilopascal-seconds; 1/kPa·s, reciprocal kilopascal-seconds; mmol/min/kPa, millimoles/minute/kilopascal; mmol/min/kPa/L, millimoles/minute/kilopascal/litre; g/dL, grams/decilitre.

Pulmonary function testing demonstrated restrictive ventilatory impairment, with total lung capacity (TLC) of 62%, forced expiratory volume in one second (FEV1) of 52%, FEV1/forced vital capacity (FVC) ratio of 76%, maximal vital capacity of 47%, and diffusing capacity for carbon monoxide (DLCO) of 63% (Figure 2A, 2B).

**Figure 3:**
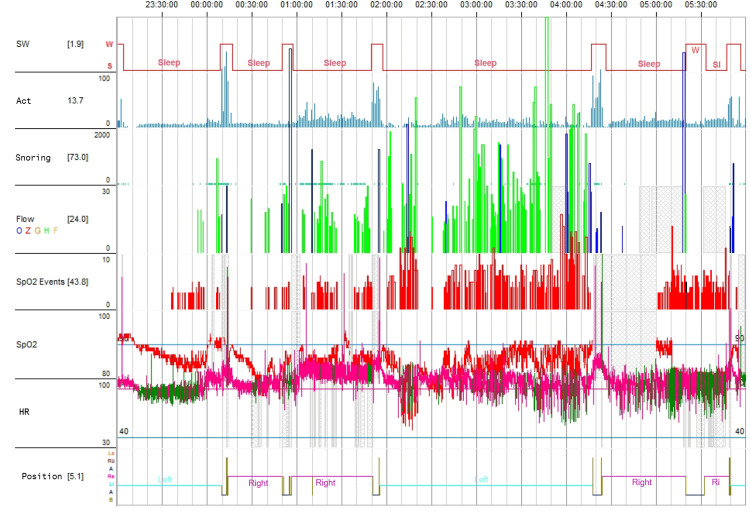
Representative polygraphy tracing showing recurrent respiratory events with oxygen desaturations and snoring during sleep. Abbreviations: SW, sleep-wake state; W, wake; S, sleep; Akt, activity; Snoring, snoring signal; Flow, respiratory flow signal; O, obstructive apnoea; Z, central apnoea; G, mixed apnoea; H, hypopnoea; F, flow limitation; SpO2 Events, oxygen desaturation events; SpO2, peripheral oxygen saturation; HR, heart rate; Position, body position; RU, supine position; Re, right lateral position; Li, left lateral position; A, upright position; B, prone position. Units: SpO2, peripheral oxygen saturation (%); HR, heart rate (bpm); time, hours:minutes:seconds.

Sleep study with polygraphy demonstrated moderate lateral position-dependent obstructive sleep apnoea with an apnoea-hypopnoea index (AHI) of 25 events per hour and severe nocturnal hypoxaemia with an oxygen desaturation index (ODI) of approximately 45 events per hour (mean oxygen saturation 84%, nadir approximately 65%) (Figure 3).

Arterial blood gas analysis on room air demonstrated metabolic alkalosis with an elevated PaCO₂, with a pH of 7.51, bicarbonate (HCO₃⁻) of 41.5 mmol/L (reference range 21.0-26.0 mmol/L), partial pressure of carbon dioxide (PaCO₂) of 6.9 kPa (reference range 4.7-6.1 kPa), and partial pressure of oxygen (PaO₂) of 9.9 kPa (reference range 9.5-13.9 kPa). Potassium was 2.2 mmol/L (reference range 3.5-5.1 mmol/L) and ionised calcium was transiently low at 0.93 mmol/L (reference range 1.15-1.27 mmol/L), the latter being the lowest recorded value.

**Figure 4:**
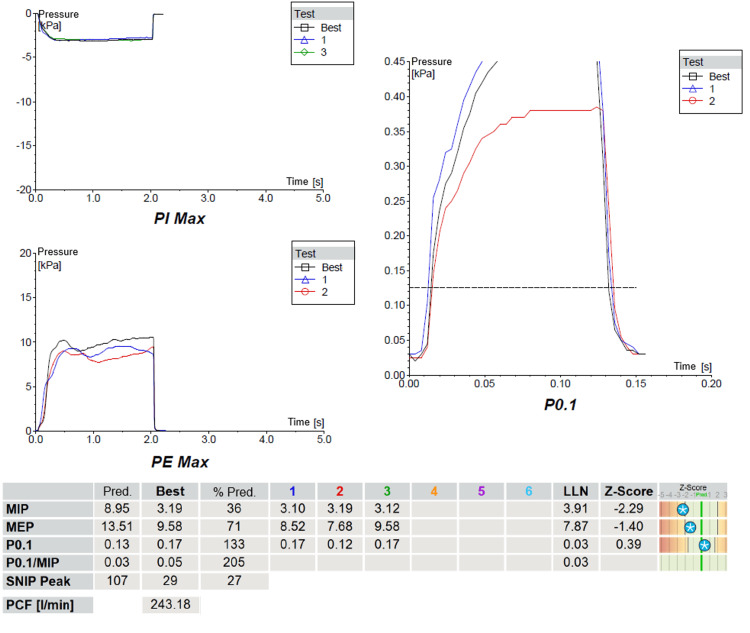
Respiratory muscle testing showing markedly reduced inspiratory muscle strength, with low MIP and SNIP, relatively preserved MEP, and elevated P0.1. Abbreviations: PImax/MIP, maximal inspiratory pressure; PEmax/MEP, maximal expiratory pressure; P0.1, airway occlusion pressure 0.1 seconds after onset of inspiration; P0.1/MIP, ratio of airway occlusion pressure to maximal inspiratory pressure; SNIP Peak, peak sniff nasal inspiratory pressure; PCF, peak cough flow; Pred., predicted; Best, best recorded value; % Pred., percentage of predicted value; 1, 2, 3, 4, 5, 6, individual manoeuvre attempts; LLN, lower limit of normal; Z-score, z score relative to the reference population; Units: kPa, kilopascals; L/min, litres per minute; s, seconds.

Respiratory muscle testing demonstrated marked inspiratory muscle weakness with a maximal inspiratory pressure (MIP) of 36% predicted and sniff nasal inspiratory pressure (SNIP) of 27% predicted. Expiratory strength was relatively preserved with maximal expiratory pressure (MEP) of 71% predicted. Airway occlusion pressure at 0.1 seconds (P0.1) was elevated at 133% predicted, indicating increased respiratory drive. These findings were consistent with ventilatory pump limitation and supported suspected diaphragmatic dysfunction (Figure 4).

Echocardiography showed normal left ventricular systolic function (LVEF 65%-70%) and no evidence of pulmonary hypertension. Neurological examination in the supine position demonstrated paradoxical breathing with inspiratory elevation of the thorax and simultaneous inward abdominal movement, supporting suspected diaphragmatic dysfunction. Endocrinology consultation considered the profound hypomagnesaemia multifactorial, likely related to diarrhoea and chronic proton pump inhibitor use. Urinary indices suggested renal potassium loss. Differential diagnoses included primary aldosteronism and renal tubular disorders such as Gitelman syndrome.

**Figure 5:**
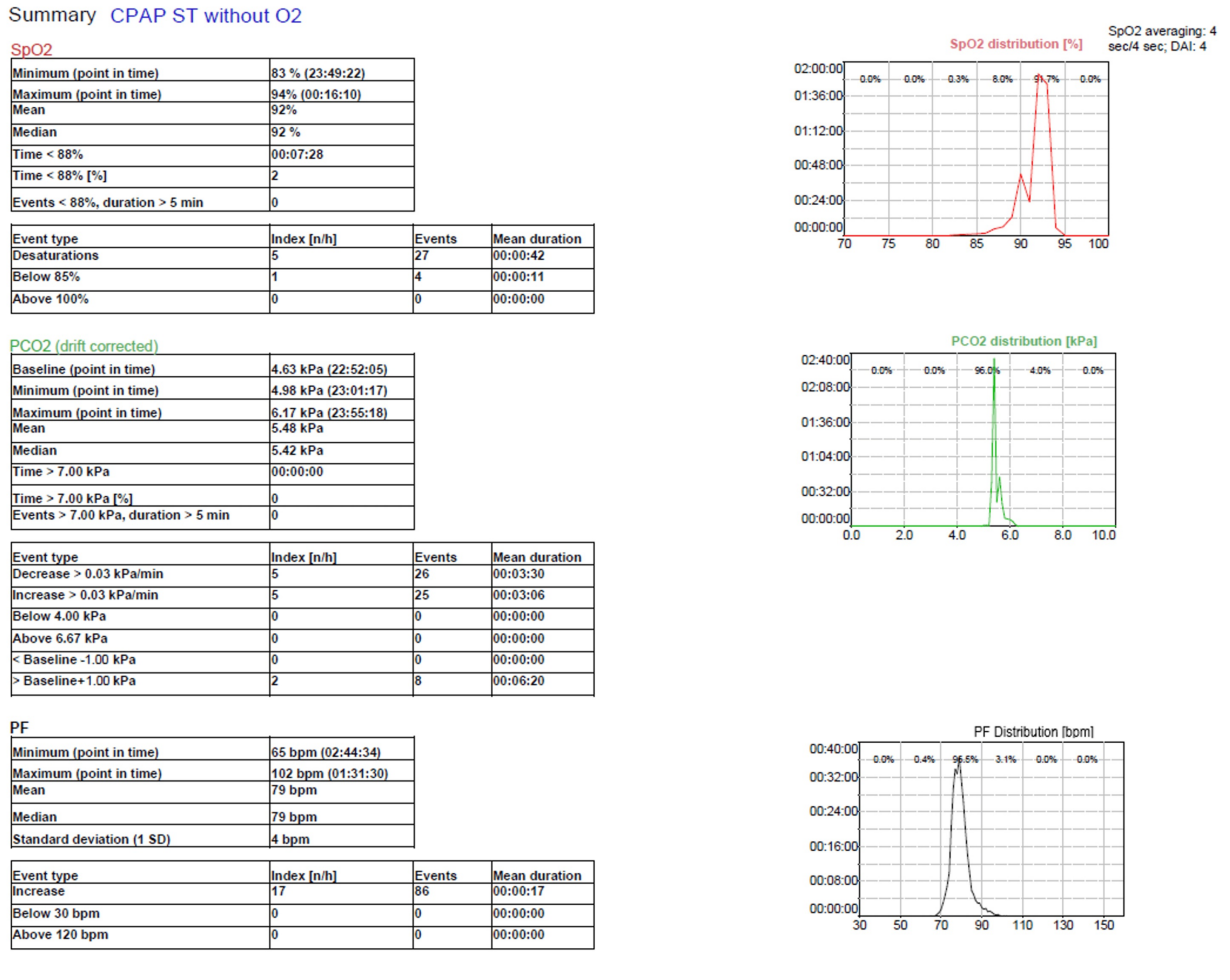
Overnight transcutaneous capnography during CPAP therapy showing stable nocturnal oxygenation and transcutaneous carbon dioxide levels without sustained hypercapnia. CPAP, continuous positive airway pressure; SpO2, peripheral oxygen saturation; PCO2, transcutaneous partial pressure of carbon dioxide; PF, pulse frequency; DAI, desaturation area index; Index [n/h], event index in number per hour; Mean duration, mean event duration; bpm, beats per minute. Units: kPa, kilopascals; bpm, beats per minute; n/h, number per hour; min, minutes; s, seconds; h:min:s, hours:minutes:seconds.

Nocturnal CPAP was initiated for sleep-disordered breathing and symptomatic orthopnoea, resulting in rapid improvement in the ability to sleep recumbent. Overnight transcutaneous capnography during CPAP therapy demonstrated stable nocturnal oxygenation and no sustained hypercapnia (Figure 5). Magnesium and potassium were corrected and supplemented orally. Proton pump inhibitor therapy was discontinued.

The patient was discharged with CPAP therapy, and outpatient neurological and endocrine follow-up was planned. Approximately one month after discharge, he died unexpectedly from a fulminant myocardial infarction. Definitive neuromuscular and endocrine investigations could not be completed.

## Discussion

This case illustrates that severe orthopnoea may already indicate ventilatory pump failure even when cardiopulmonary imaging, including CT and echocardiography, is unrevealing. There was no evidence of heart failure, pulmonary embolism, interstitial lung disease, or central airway obstruction, making a primarily neuromuscular cause more likely. The defining clinical feature was immediate dyspnoea in the supine position with paradoxical breathing, a classical sign of diaphragmatic weakness [1]. Restrictive spirometry, marked inspiratory muscle weakness on respiratory muscle testing, and elevated PaCO₂ in the context of metabolic alkalosis supported ventilatory pump limitation [1,2,8]. The degree of nocturnal desaturation appeared disproportionate to the severity of obstructive sleep apnoea and suggested reduced ventilatory reserve rather than isolated upper airway obstruction [2,8]. CPAP likely improved upper airway patency and stabilised nocturnal ventilation, explaining the rapid symptomatic response despite the unresolved underlying mechanism. Severe hypomagnesaemia and hypokalaemia may have contributed to diaphragmatic dysfunction [1,5], but persistence of paradoxical breathing after electrolyte correction argued against an electrolyte disturbance as the sole explanation.

Other systemic features were considered but were not felt to provide a unifying explanation. The chronic productive cough may in part be explained by the patient’s ongoing tobacco use, although this does not fully exclude additional pulmonary pathology. The reported weight loss had been intentional according to the patient. These findings were therefore considered of uncertain relevance to the ventilatory disorder, while not entirely excluding the possibility of an additional systemic process.

The differential diagnosis therefore focused primarily on neurological causes. Compressive or inflammatory pathology affecting the cervical spinal cord, particularly at C3-C5, may impair respiratory pathways and lead to diaphragmatic paresis [9]. Isolated phrenic neuropathy also represents an important cause of diaphragmatic dysfunction. Potential mechanisms include mechanical compression, such as tumour or cervical disc disease, as well as inflammatory neuropathies including neuralgic amyotrophy or Parsonage-Turner syndrome, which may involve the phrenic nerve and result in diaphragmatic paralysis [10]. Infectious processes such as Borrelia infection or herpes zoster, as well as rare variants of Guillain-Barré syndrome with predominant respiratory involvement, were also considered. Disorders of the neuromuscular junction, particularly myasthenia gravis, may present with isolated respiratory muscle weakness and positional dyspnoea before more generalised symptoms become apparent [2]. Primary muscle disease was likewise considered, including metabolic or genetic myopathies such as late-onset Pompe disease and inflammatory myositis. In late-onset Pompe disease, diaphragmatic involvement may rarely represent the earliest or predominant manifestation [11]. Electrolyte disturbance was therefore interpreted as a possible contributory factor rather than a sufficient unifying diagnosis because paradoxical breathing persisted despite normalisation of potassium and magnesium.

## Conclusions

Severe orthopnoea with normal cardiopulmonary imaging should prompt evaluation for ventilatory pump failure. Positional paradoxical breathing and inspiratory muscle weakness are key indicators of diaphragmatic dysfunction. Moderate obstructive sleep apnoea with nocturnal hypoxaemia that appears disproportionate to the measured severity of sleep-disordered breathing may indicate coexisting hypoventilation. CPAP can provide rapid symptomatic benefit even when the underlying neuromuscular aetiology remains unconfirmed.
